# Editorial Degradation

**Published:** 1856-07

**Authors:** 


### EDITORIAL DEGRADATION.

Mighty prompt were New York Editors within a year to raise, the hue and cry against a Judge who was tried for official corruption in this city ; and they have the holiest horror too, as to men who *have their price.* In describing the characters of those who are convicted of official corruption, the English language is depleted of its adjectival supply. But what means the following incidents ?

A paper published in a southern city, in noticing that during eighteen hundred and fifty-five, the number of deaths from all causes, was smaller than during the preceding year, intimates the belief, that this was a result of the freer use of a certain man's medicines, which medicines were largely advertised in that paper. Several of the New York dailies and weeklies have been paid to insert among their reading matter, editorials from one another, and an article from another publication, that the reason why there were fewer deaths from *Consumption* in New York during eighteen hundred and fifty-five, than during the preceding year, notwithstanding it had a larger population, was properly attributed to the fact that *Medicated Inhalation* had been introduced as a remedy, and then they launched out in praises of the same at *a dollar a line!* Now having given currency to an untruth for pay, will any one of them show a late repentance, by publishing without pay, the following statements, taken from published official tables, showing that not only in New York, where Inhalation was fabricated and cherished, but also in Philadelphia and Boston and Baltimore, which had not yet taken the infection, not only were deaths from Consumption fewer than during the previous year, but deaths from all other diseases were fewer in number. This double fact, simple enough in itself, and undeniable while it sinks beneath contempt the men who have made use of a part of a fact to bolster up a falsehood to make money thereby, at the expense of the health of their fellow citizens, does at the same time present a humiliating picture of the credulity or recklessness or purchaseability of some among us, who conduct the editorial department of our newspapers.

The mortality in four of our larger cities during the three years past is as follows:

| | 1853. | 1854. | 1855. |
|---|---|---|---|
| New York, | 21,864. | 28,458. | 23,107. |
| Philadelphia, | 9,750. | 11,811. | 10,509. |
| Baltimore, | 5,117. | 5,938. | 5,447. |
| Boston, | 4,369. | 4,418. | 4,030. |

That is to say, the rate of decrease of death from all causes in 1855, as compared with 1854, was thirteen per cent for New York, twelve per cent for Philadelphia, nine per cent for Boston and three per cent for Baltimore.

We think that the unfairness in making use of a part of a fact, must lower the authorship, as well as the abettors of the same, in the estimation of all lovers of truth. It is the exaggeration of this, which makes the common liar and the heartless perjurer. And it must be apparent to every person of reflection that any system which needs such aid cannot be true, that any system which draws to it men who are reckless of the truth, must be as baseless as the characters of its advocates.

